# Food for thought: association between dietary tyrosine and cognitive performance in younger and older adults

**DOI:** 10.1007/s00426-017-0957-4

**Published:** 2017-12-18

**Authors:** Simone Kühn, Sandra Düzel, Lorenza Colzato, Kristina Norman, Jürgen Gallinat, Andreas M. Brandmaier, Ulman Lindenberger, Keith F. Widaman

**Affiliations:** 1 Center for Lifespan Psychology, Max Planck Institute for Human Development, Berlin, Germany; 2 Clinic and Policlinic for Psychiatry and Psychotherapy, University Clinic Hamburg-Eppendorf, Hamburg, Germany; 3 Institute for Brain and Cognition and Department of Psychology, University of Leiden, Leiden, The Netherlands; 4 Geriatrics Research Group, Charité University Medicine, Berlin, Germany; 5 University of California at Riverside, University of California, California, USA

## Abstract

The fact that tyrosine increases dopamine availability that, in turn, may enhance cognitive performance has led to numerous studies on healthy young participants taking tyrosine as a food supplement. As a result of this dietary intervention, participants show performance increases in working memory and executive functions. However, the potential association between habitual dietary tyrosine intake and cognitive performance has not been investigated to date. The present study aims at clarifying the association of episodic memory (EM), working memory (WM) and fluid intelligence (Gf), and tyrosine intake in younger and older adults. To this end, we acquired habitual tyrosine intake (food frequency questionnaire) from 1724 participants of the Berlin Aging Study II (1383 older adults, 341 younger adults) and modelled its relations to cognitive performance assessed in a broad battery of cognitive tasks using structural equation modeling. We observed a significant association between tyrosine intake and the latent factor capturing WM, Gf, and EM in the younger and the older sample. Due to partial strong factorial invariance between age groups for a confirmatory factor analysis on cognitive performance, we were able to compare the relationship between tyrosine and cognition between age groups and found no difference. Above and beyond previous studies on tyrosine food supplementation the present result extend this to a cross-sectional association between habitual tyrosine intake levels in daily nutrition and cognitive performance (WM, Gf, and EM). This corroborates nutritional recommendations that are thus far derived from single-dose administration studies.

## Introduction

The two statements “Tell me what you eat and I will tell you who you are” (“Dis-moi ce que tu manges, je te dirai ce que tu es”) (Brillat-Savarin, 1826/1842) by the French epicure and gastronome Anthelme Brillat and “Man is what he eats” (“Der Mensch ist, was er ißt”) by the German philosopher Ludwig Feuerbach have become veritable proverbs in Western societies in which healthy nutrition is an increasingly important concern. The idea that food influences the way we think, feel, and act has become very popular (illustrated by cookbooks with names such as “Save Your Brain Cookbook”, “The Healthy Mind Cookbook”). In addition, indeed, some amino acids, which are the building blocks of proteins in our daily food, constitute precursors of neurotransmitters that can alter brain function. Within the organism, dietary L-tyrosine (termed, more simply, tyrosine from here onwards) is converted by the enzyme hydroxylase into L-Dopa, the direct precursor of dopamine, which in turn is converted to norepinephrine (Fernstrom & Fernstrom, 2007). Because hydroxylase is commonly about 75% saturated with tyrosine, higher tyrosine levels may have the potential to increase the brain’s dopamine and norepinephrine synthesis (Carlsson & Lindqvist, 1978). Foods high in dietary tyrosine include cheese, soybeans, beef, lamb, pork, fish, chicken, nuts, eggs, dairy, beans, and whole grain.

The neurotransmitter dopamine has long been implicated in cognitive processes such as working memory (Bäckman, Nyberg, Lindenberger, Li, & Farde, 2006) as well as learning and reward processing (Beninger & Miller, 1998) and is known to play an important role in aging (Bäckman, Lindenberger, Li, & Nyberg, 2010). The fact that tyrosine increases dopamine availability that, in turn, may enhance cognitive performance has led to numerous studies administering tyrosine as a food supplement. In most of these studies, only one dose of tyrosine was administered before cognition was tested (for an overview, see Jongkees, Hommel, Kühn, & Colzato, 2015; Hase, Jung, & aan het Rot, 2015; van de Rest, van der Zwaluw, & de Groot, 2013). Even in response to single doses (ranges of 100–300 mg/kg body weight) of tyrosine supplements, effects on cognition have been shown, in particular on working memory performance (Colzato, Jongkees, Sellaro, & Hommel, 2013; Deijen & Orlebeke, 1994; Deijen, Wientjes, Vullinghs, Cloin, & Langefeld, 1999; Magill et al., 2003; Mahoney, Castellani, Kramer, Young, & Lieberman, 2007; O’Brien, Mahoney, Tharion, Sils, & Castellani, 2007; Shurtleff, Thomas, Ahlers, & Schrot, 1993; Thomas, Lockwood, Singh, & Deuster, 1999), but initial evidence also exists for the influence on executive functions such as cognitive flexibility (Steenbergen, Sellaro, Hommel, & Colzato, 2015; Deijen & Orlebeke, 1994), inhibition (Colzato, Jongkees, Sellaro, van den Wildenberg, & Hommel, 2014), convergent thinking (Colzato, de Haan, & Hommel, 2015), and reasoning (Magill et al., 2003).

The literature to date suggests that tyrosine is most effective in cases of neurotransmitter depletion, namely, when dopamine and norepinephrine levels are reduced. This is the case, for example, when the organism is exposed to stress such as hypothermia or a cognitively challenging task, and therefore, an increasing number of neurotransmitters are synthesised (Kvetnansky, Sabban, & Palkovits, 2009) or at older ages (Bäckman et al., 2010). Studies investigating the cognitive effects of long-term tyrosine intake are rare. A randomized placebo-controlled study on residents of Antarctica focussed on mood and found improvements during the winter (Palinkas et al., 2007), but did not report effects on cognitive performance. Unfortunately, research on the effects of habitual tyrosine intake in daily nutrition (including, for example, foods that are high in tyrosine, such as soy, eggs, and salmon) is lacking. We aimed to fill this gap in the literature by investigating the association between habitual tyrosine intake, derived from a food frequency questionnaire, with cognitive performance assessed in a broad battery of cognitive tasks using structural equation modeling in both younger and older adults. Based on the previous studies investigating the enhancing effects of a single dose of tyrosine supplements, we hypothesized a positive association between a diet rich in tyrosine and both working memory performance and executive functions. Moreover, based on the fact that dopamine transmission is reduced in older age, we expected a stronger association between tyrosine and cognitive performance in older compared with younger adults.

## Methods

### Participants and study design

Participants were recruited within the Berlin Aging Study II (BASE-II) (for cohort characteristics and additional details, see Bertram et al., 2014; Gerstorf et al., 2016). The sample used in the present study comprised 1724 participants (the original sample consists of 2200 subjects, but we reduced the sample to those of which we have food intake information). Of these, 1383 were older adults aged 61–88 years (mean age 70.7, SD 3.89; 692 female), and 341 were younger adults aged 24–40 years (mean age 31.1, SD 3.38; 182 female). On average, older participants had 14.59 years of education (SD 3.03), and younger participants 15.53 years (SD 2.47). None of the participants was on medication that may have affected memory function or had a history of head injuries, medical (e.g., heart attack), neurological (e.g., epilepsy), or psychiatric disorders (e.g., depression).

### Cognitive assessment

The cognitive battery of BASE-II included the assessment of multiple tasks. Here, we focus on three main cognitive abilities: episodic memory (EM; indicated by Verbal Learning and Memory Test, Face–Profession Task, and Scene Encoding), working memory (WM; indicated by Letter Updating, Number-N-Back, and Spatial Updating), and fluid intelligence (Gf; indicated by Figural Analogies, Letter Series, and Practical Problems). The chosen tasks varied in procedures and content, consisting of items that relate to verbal, numerical, or figural–spatial information (for more information, refer to Duzel et al., 2016).

### Nutrition assessment

We used the European Prospective Investigation into Cancer and Nutrition (EPIC) food frequency questionnaire (FFQ) to assess daily average dietary tyrosine intake in grams (Boeing, Bohlscheid-Thomas, Voss, Schneeweiss, & Wahrendorf, 1997). The questionnaire assesses dietary intake of 148 foods during the last 12 months. For each item and given portion size (indicated by means of line drawings), participants rate the frequency of consumption. Then, we derived the nutrient concentrations for each food item of the FFQ by taking values provided by the Federal Coding System. In our analyses, we used the average amount of tyrosine and the average amount of food consumed per day.

### Structural equation modeling

We used structural equation modeling (SEM) to investigate the relations between tyrosine and cognitive performance for two reasons. First, SEM enables us to move beyond modeling at the manifest variable level to modeling the cognitive variables at the construct level, namely, on latent variables for working memory, episodic memory, and fluid intelligence. In doing so, we can account for and partial out the effects of measurement error, which enhances the validity of our analyses (Little, Lindenberger, & Nesselroade, 1999). Second, SEM offers a generic framework to formalize and test our hypotheses about the potential interrelations of cognition and nutrition. Our focal research question was whether dietary tyrosine affects cognitive performance in younger and in older adults. Due to the cross-sectional and observational nature of this data set, we cannot clarify the direction of effects. Because the BASE-II sample consists primarily of older participants, but also includes a smaller subsample of younger participants, we aimed to address the question whether the relations between cognition and tyrosine is comparable across the two age groups by means of a multi-group SEM analysis.

To ascertain the interpretability of quantitative group comparisons, we needed to establish measurement invariance, that is, evaluate whether the latent constructs fall on the same scale across groups. The prerequisites for this comparison are configural invariance, weak invariance, and strong invariance across the groups, and these levels of invariance are tested by making increasingly restrictive assumptions in different models. Configural invariance is satisfied when the basic measurement model structure (i.e., the pattern of fixed and free loadings of manifest variables on latent variables) is the same across groups. Weak invariance involves constraining factor loadings to invariance across groups, which ensures equal metrics on the latent variables across groups. Strong invariance additionally requires the intercepts of the indicators to be the same. In addition, one can test for strict invariance, which constrains residual variances of indicators to be invariant across age groups, that is, measures are assumed to be equally reliable across groups. However, there is general consensus that strict invariance need not be established for comparing means and (co)variances on the latent variable level. These invariance levels were tested in hierarchical order based on model fit indices. Invariance was tested based on the recommendations of Widaman and Reise (1997) and Cheung and Resvold (2002) (but see McCallum, Browne, & Cai, 2006, for an alternate approach to model comparison).

We report the Chi-square goodness of fit index for all models, but this statistical index often suggests rejection of close fitting models when sample size is large, as was the case in this study. Therefore, we relied on standard indices such as the root mean square error of approximation (RMSEA), standardized root mean square residual (SRMR), the comparative fit index (CFI), the Tucker–Lewis index (TLI), and the Bayesian information criterion (BIC) for evaluation of model fit. Commonly accepted thresholds indicating good model fit are RMSEA ≤ 0.05, SRMR ≤ 0.05, and CFI and TLI ≥ 0.95, and a lower BIC implies better fit (Schermelleh-Engel, Kerwer, & Klein, 2014; Hu & Bentler, 1999). The analyses were run using MPlus v6.1 (Muthen & Muthen, 2010).

**Fig. 1:**
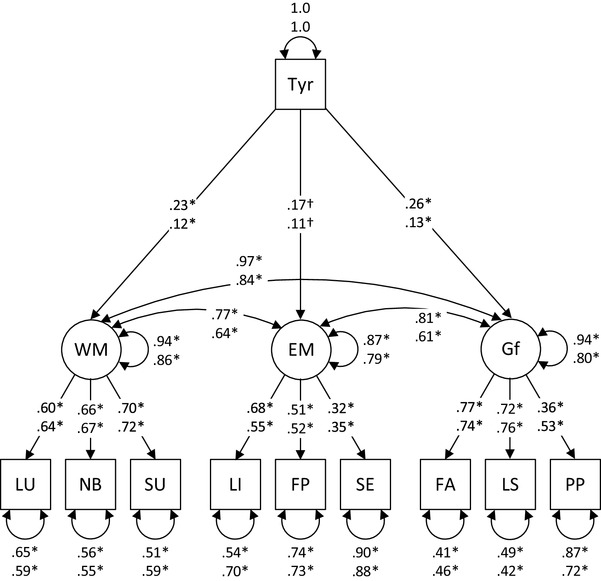
Simplified illustration of the structural equation model depicting effects of tyrosine (Tyr) on the latent cognitive factors for working memory (WM), episodic memory (EM), and fluid intelligence (Gf). For each path, the top number is for the young adult group and the bottom number is for the older adult group. Numbers next to single-headed solid arrows represent significant regression coefficients. Numbers next to double-headed arrows represent standardized variances or covariances. *p < 0.001. ^†^*p* = 0.02

Next, we used latent regression analyses to investigate how EM, WM, and Gf relate to tyrosine intake as a function of age (Fig. 1). Missing data were handled using a full information maximum likelihood approach as implemented in the Mplus software. We set up a multi-group structural equation model to test whether associations between the three-factor model of cognition and tyrosine intake varied across age groups. In addition, four covariates were entered to the SEM as exogeneous predictors of cognition and tyrosine: sex, age, years of formal education, and overall food intake.

To test from which sources participants most likely boost their tyrosine levels, we correlated dietary tyrosine levels with the different food categories according to which foods are grouped in the EPIC food frequency questionnaire (potatoes, vegetables, legumes, fruits, dairy, cereal products, meat products, fish, egg products, fat, sugar, cake, spices and sauces, and soups) while controlling for overall food intake.

## Results

There was no significant difference in daily tyrosine intake between the age groups, *t*(1717) = 1.20, *p* = 0.23. Younger participants consumed an average of 2.85 g of tyrosine daily (SD 1.36, range 1–17 g) and older participants 2.77 g (SD 0.946, range 1–10). Likewise, the average food intake (in g) did not differ between the age groups, *t*(1722) = − 1.59, *p* = 0.11, with younger participants consuming 1345 g (SD 413) and older participants consuming 1383 g per day (SD 390). However, we did observe a significant difference in body mass index (younger participants: mean = 23.39, SD 4.26, older participants: mean = 26.82, SD 4.11, *t*(1712) = − 13.64, *p* < 0.001). In cognitive performance, strong differences were observed between age groups in all test scores, with younger outperforming older participants (see Table 1).

**Table 1:** Group differences in cognitive performance between the two age groups

Cognitive task	Younger participants mean (SD)	Older participants mean (SD)	Group difference (*t* value)
Letter updating (LU)	46.96 (9.80)	38.78 (11.37)	13.24**
Number-N-back (NB)	0.89 (0.13)	0.68 (0.18)	24.60**
Spatial updating (SU)	33.24 (6.79)	20.77 (9.42)	27.74**
Verbal learning and memory test (LI)	12.13 (2.09)	8.48 (2.69)	27.05**
Face profession (FP)	0.55 (0.19)	0.26 (0.14)	23.91**
Scene encoding (SE)	0.38 (0.15)	0.28 (0.14)	12.15**
Figural analogies (FA)	17.15 (3.19)	11.52 (5.17)	25.18**
Letter series (LS)	16.19 (4.97)	9.04 (6.30)	22.30**
Practical problems (PP)	12.45 (2.88)	10.29 (2.76)	12.49**

**p < 0.001

### CFA of cognitive performance indicators across groups

Confirmatory factor analysis (CFA) was conducted to define the three latent factors of individual differences in WM, EM, and Gf across both age groups. The first model was a configural invariance model, with the same pattern of fixed and free factor loadings across groups. This model had a significant Chi-square index, *χ*^2^ (48) = 125.75, *p* < 0.001, but acceptable fit to the data, with CFI = 0.979, TLI = 0.968, RMSEA = 0.043, and BIC = 56184.2.

The next model estimated the weak factorial invariance model with invariant factor loadings across the younger adult and older adult groups. This model also had a significant Chi-square index, *χ*^2^ (54) = 155.97, *p* < 0.001, and the change in statistical fit was statistically significant, ∆*χ*^2^ (6) = 30.22, *p* < 0.001. However, the practical fit indices were altered little, with CFI = 0.972, TLI = 0.962, and RMSEA = 0.047, and the BIC = 56169.7 was lower, indicating that this model had better fit to the data than did the configural model.

The third model was the strong factorial invariance model, which imposed cross-group invariance constraints on measurement intercepts. The strong factorial invariance model fit decidedly worse than the weak invariance model, *χ*^2^ (60) = 224.47, *p* < 0.001, and the change in statistical fit was significant, and the change in fit was large, ∆*χ*^2^ (6) = 68.50, *p* < 0.001. Moreover, the practical fit indices were noticeably worse, with CFI = 0.955, TLI = 0.945, and RMSEA = 0.056, and the BIC = 56193.5 increased, demonstrating poor fit of this model. Based on modification indices, we relaxed one intercept invariance constraint on the Letter Updating variable resulting in a partial strong invariance model, which exhibited considerably improved fit, with *χ*^2^ (59) = 169.09, *p* < 0.001, and a large improvement in fit over the full strong invariance model, ∆*χ*^2^ (1) = 55.38, *p* < 0.001. The practical fit indices were much improved, with CFI = 0.970, TLI = 0.963, and RMSEA = 0.046, and the BIC = 56145.6 was the lowest of all models considered to this point, suggesting that the partial strong invariance model is tenable and is the optimal model for these data.

Parameter estimates for the partial strong factorial invariance model are shown in Table 2, which provides the raw score estimates of all parameters. As shown in Table 2, the model was identified by fixing factor means to 0.0 and factor variances to 1.0 in the younger adult group, estimating factor correlations for the younger adult group, constraining factor loadings and intercepts to invariance across groups, and estimating factor means, variances, and covariances in the older adult group. All parameter estimates were significant at *p* < 0.0001. Standardized factor loadings were in the moderate-to-strong range, with median standardized loadings of 0.65 in each group. As shown in Table 2, the WM and Gf factors were highly correlated in both samples, and correlations of the EM factor with the WM and Gf factors were weaker, but still large in magnitude, as predicted.

**Table 2:** Multiple-group confirmatory factor analysis for nine cognitive performance variables for young and older adults

Variable	Intercept	Factor loadings			Unique variance	
		WM	EM	Gf	Young	Old
Letter updating	46.9 (0.51)^a^	5.70 (0.37)	0.0^c^ (0.00)	0.0^c^ (0.00)	53.8 (4.84)	77.9 (3.59)
Number-N-back	0.89 (0.01)	0.09 (0.01)	0.0^c^ (0.00)	0.0^c^ (0.00)	0.01 (0.001)	0.02 (0.001)
Spatial updating	33.2 (0.37)	5.08 (0.29)	0.0^c^ (0.00)	0.0^c^ (0.00)	24.9 (2.51)	44.0 (2.26)
VLMT^b^	12.1 (0.11)	0.0^c^ (0.00)	1.38 (0.11)	0.0^c^ (0.00)	2.35 (0.28)	5.02 (0.25)
Face profession	0.54 (0.01)	0.0^c^ (0.00)	0.10 (0.01)	0.0^c^ (0.00)	0.03 (0.003)	0.03 (0.001)
Scene encoding	0.40 (0.01)	0.0^c^ (0.00)	0.05 (0.01)	0.0^c^ (0.00)	0.02 (0.002)	0.02 (0.001)
Figural analogies	17.1 (0.18)	0.0^c^ (0.00)	0.0^c^ (0.00)	2.57 (0.15)	4.63 (0.53)	11.3 (0.61)
Letter series	16.2 (0.24)	0.0^c^ (0.00)	0.0^c^ (0.00)	3.28 (0.19)	11.2 (1.09)	16.3 (0.93)
Practical problems	12.4 (0.11)	0.0^c^ (0.00)	0.0^c^ (0.00)	0.99 (0.07)	7.16 (0.56)	5.50 (0.23)

	Factor					
	Mean	Factor covariances				
Young adult						
Factor						
WM	0.0^c^ (0.00)	1.0^c^ (0.00)	0.77	0.97		
EM	0.0^c^ (0.00)	0.77 (0.07)	1.0^c^ (0.00)	0.80		
Gf	0.0^c^ (0.00)	0.97 (0.04)	0.80 (0.06)	1.0^c^ (0.00)		
Older adult						
Factor						
WM	− 2.43 (0.16)	1.68 (0.21)	0.67	0.86		
EM	− 2.65 (0.22)	0.94 (0.12)	1.16 (0.20)	0.65		
Gf	− 2.18 (0.15)	1.67 (0.18)	1.04 (0.13)	2.24 (0.28)		

Tabled values are parameter estimates, with standard errors in parentheses. All parameter estimates had critical ratios > 5.8, so were significant at *p* < 0.0001. In factor covariance matrices, factor variances are on the diagonal, and covariances are shown below the diagonal and correlations above the diagonal

^a^Intercept for older adult sample = 52.7 (SE = 0.75)

^b^*VLMT* verbal learning and memory test

^c^Values fixed at reported values to identify model

The most important pattern in the results was the large mean differences on latent factors across groups. Because the model was identified with latent variables means of 0 and SDs of 1.0 in the younger adult sample, the mean differences on latent factors for the older group were in a Cohen’s *d* metric. The mean differences in performance showed that the older adult group scored more than 2 SD units below the younger adult group on all three factors, with mean differences of − 2.43 (SE = 0.16) on the WM factor, − 2.65 (SE = 0.22) on the EM factor, and − 2.18 (SE = 0.15) on the Gf factor. Thus, the mean differences in performance across groups were substantial.

### Associations with tyrosine

We hypothesized that individuals’ tyrosine intake would be linked to their current cognitive status. In this model, our primary predictor of performance on the cognitive factors (WM, EM, and Gf) was tyrosine intake, and sex, education, age, and average food intake were control variables. The effects of tyrosine intake, sex, education, age, and average food intake on the cognitive factors were allowed to vary across the young adult and old adult groups, so we termed this model an initial unconstrained model. The fit of this model, which retained the partial strong invariance measurement constraints on cognitive factors, was acceptable. The Chi-square index was significant, *χ*^2^ (124) = 262.35, *p* < 0.001, but the model exhibited close fit to the data, with CFI = 0.976, TLI = 0.965, RMSEA = 0.036, and BIC = 80493.9.

We then constrained the path coefficients from tyrosine to the cognitive factors and average food intake to the cognitive factors to invariance across the younger adult and older adult groups to test whether effects of tyrosine and food intake on cognition differed across groups. The cross-group invariance constraints on effects of tyrosine and food intake on Gf led to a non-significant change in model fit, ∆*χ*^2^ (2) = 3.59, *p* = 0.17. Invariance constraints on effects of tyrosine and food intake on WM were also non-significant, ∆*χ*^2^ (2) = 0.50, *p* = 0.64. Invariance constraints on effects of tyrosine and food intake on EM were also non-significant, ∆*χ*^2^ (2) = 0.89, *p* = 0.91. Finally, the omnibus constrained model invoked cross-group invariance constraints of tyrosine and food intake simultaneously on all three cognitive factors. This model had a significant statistical index of fit, with *χ*^2^ (130) = 271.54, *p* < 0.001, but the change in fit from the initial unconstrained model was small and non-significant, ∆*χ*^2^ (6) = 9.19, *p* = 0.30. Moreover, the practical fit indices were either unchanged or improved, with CFI = 0.975, TLI = 0.966, and RMSEA = 0.035. Finally, the BIC = 80458.4 was lower (i.e., improved), indicating that the cross-group invariance constraints on parameter estimates did not harm model fit. We thus accepted this model as the final model for the data.

Standardized parameter estimates from our final model are shown in Fig. 1. Inspection of Fig. 1 reveals that factor loadings in the young adult sample were in the moderate-to-strong range (median loading = 0.66) and were of similar magnitude in the older adult sample (median loading = 0.64). We found significant direct effects of tyrosine on Gf in the young adult sample, *β* = 0.26 (SE = 0.07), *p* < 0.001, and in the older adult sample, *β* = 0.13 (SE = 0.04), *p* < 0.001. Tyrosine also had significant effects on WM in the young adult sample, *β* = 0.23 (SE = 0.07), *p* < 0.001, and in the older adult sample, *β* = 0.12 (SE = 0.04), *p* < 0.001. The effects of tyrosine on EM were also significant, in the young adult sample, *β* = 0.17 (SE = 0.07), *p* = 0.02, and in the older adult sample, *β* = 0.11 (SE = 0.05), *p* = 0.02. Effects of predictors explained 6% of the variance of Gf, 6% of the variance of WM, and 13% of the variance of EM in the young adult sample, and 21% of the variance in Gf, 14% of the variance in WM, and 21% of the variance of EM in the older adult sample.

In Table 3, within-group standardized regression coefficients are reported, along with the variance explained for each ability factor in each sample. The greater variance explained in the older adult group relative to the young adult group on the three ability factors of Gf, WM, and EM is due primarily to the effects of covariates, in particular the effect of education. In the young adult group, the direct effect of education explained between 2% (0.15 squared = 0.023, for EM) and 3% (0.18 squared = 0.032, for Gf) of the variance of the three ability factors. In contrast, the direct effect of education explained between 9.6% (0.31 squared = 0.096, for WM) and 15% (0.39 squared = 0.152, for Gf) of the variance of the three ability factors. The positive signs of the regression weights for education reflect the tendency for persons with higher levels of education to have higher scores on ability dimensions. In addition, the much stronger coefficients for education in the older adult sample are consistent with prior research investigating education as a proxy for cognitive reserve, which counteracts the negative effects of aging of human abilities. Inspection of Table 3 will also show that the effect of age on ability factors was very weak in the young adult sample, but relatively much stronger in the older adult sample, with the negative sign of these age coefficients consistent with widely reported aging declines on ability dimensions in old age. Thus, the higher levels of explained variance in the older adult sample is primarily due to the much stronger effects of education and moderately stronger effects of age on the ability factors in the older adult sample relative to the young adult sample.

**Table 3:** Standardized regression weights when regressing ability factors on covariates, food intake, and tyrosine: by sample

Sample	Predictor	Ability factor		
		Gf	WM	EM
Young	Sex	− 0.10 (0.07)	− 0.17 (0.07)	− 0.29 (0.07)
	Education	0.18 (0.06)	0.16 (0.06)	0.15 (0.07)
	Age	− 0.02 (0.05)	0.01 (0.05)	− 0.14 (0.06)
	Food intake	− 0.16 (0.05)	− 0.18 (0.05)	− 0.13 (0.05)
	Tyrosine	0.26 (0.07)	0.23 (0.07)	0.17 (0.07)
	*R* ^2^	0.06	0.06	0.13
Older	Sex	0.05 (0.03)	0.04 (0.03)	− 0.19 (0.04)
	Education	0.39 (0.03)	0.31 (0.03)	0.35 (0.04)
	Age	− 0.16 (0.02)	− 0.15 (0.02)	− 0.25 (0.03)
	Food intake	− 0.11 (0.04)	− 0.12 (0.04)	− 0.12 (0.05)
	Tyrosine	0.13 (0.04)	0.12 (0.04)	0.11 (0.05)
	*R* ^2^	0.21	0.14	0.21

Total *N* = 1728; *n* young = 343, *n* older = 1385. Tabled values are standardized regression weights, with standard errors in parentheses, and the explained variance, *R*^2^, for each factor

*Gf* fluid intelligence, *WM* working memory, *EM* episodic memory

### Exploring the categories of dietary intake providing tyrosine

In general, tyrosine intake was most strongly associated with participants’ amount of reported meat product intake (young: *r* (338) = 0.868, *p* < 0.001, old: *r* (1375) = 0.851, *p* < 0.001). All other correlation coefficients were of clearly smaller magnitude and below *r* = 0.30.

## Discussion

Within the scope of the present study, we set out to investigate the association between habitual tyrosine intake in daily nutrition and cognitive performance. To do so, we used data from the Berlin Aging study II, in which participants performed a broad range of cognitive tests, that enabled us to assess cognitive performance in three latent factors, namely, working memory, episodic memory, and fluid intelligence in relation with information about daily average dietary tyrosine intake in grams.

We started off by successfully establishing partial strong factorial invariance between the younger and the older age group in the confirmatory factor analysis capturing the cognitive performance structure. This enabled us to investigate further the potential similarities or differences in the association between tyrosine and cognitive performance as reflected in the latent factors of the structural equation model.

Previous studies on the effects of a single dose of tyrosine supplements revealed short-term benefits on working memory performance and executive functions (Jongkees et al., 2015; Hase et al., 2015; van de Rest et al., 2013). These findings are based on the knowledge that brain tyrosine levels can be modified by nutritional intake and that these concentrations and the availability to neurons also modifies the synthesis rates and release of dopamine that tyrosine is a precursor for (Fernstrom, 1983).

In line with these prior findings from intervention studies in which single doses of tyrosine were administered, we found a significant association between habitual daily tyrosine intake and performance on the latent Gf factor capturing fluid intelligence and the WM factor capturing working memory capacity. The association between tyrosine and episodic memory was also significant, but weaker.

The benefits of the present approach lie in the fact that we associate cognitive performance on a latent variable level, which enables us to purge measurements from task-specific measurement errors. Moreover, the present approach allowed us to compare the structural model between our younger and older sample and revealed no significant differences in the structure of the latent factor models and the associations of ability dimensions with tyrosine. This illustrates that the association between tyrosine and cognitive performance as measured on the latent level is comparable in younger and older adults. To our knowledge, no previous studies have investigated the effects of tyrosine in elderly or cognitively impaired participants using either dose administration or cross-sectional association approaches. Our findings suggest that the association between habitual tyrosine intake and cognitive performance is the same in younger as well as in older participants. This may at first seem surprising given that research to date suggests that acutely administered tyrosine is most effective in situations in which the organism is depleted of dopamine and/or norepinephrine (Kvetnansky et al., 2009), for example, in stressful situations such as the cold-stress test (Mahoney et al., 2007; Shurtleff et al., 1994), in a multitasking context (Thomas et al., 1999; Colzato et al., 2013) or when loud noise stimuli (Deijen & Orlebeke, 1994) are used to interfere with performance. However, older age is likewise a condition in which dopamine is depleted. Most studies report a negative relation between age and dopamine neurotransmission (Reeves, Bench, & Howard, 2002), with some studies even showing a curvilinear trajectory with enhanced losses in very-old age (Bäckman et al., 2010) and animal studies pointing at a specific decline in dopamine level within the prefrontal cortex (Arnsten, Cai, Steere, & Goldman-Rakic, 1995; Goldman-Rakic & Brown, 1981). Human post-mortem data indicate an age-related loss of dopamine D1 and D2 receptors of 3% per decade (Seeman et al., 1987). In positron emission tomography (PET) studies across the lifespan, regional declines between 5 and 13% have been described (Kaasinen et al., 2000). A PET study on humans has shown that dopamine release is modulated in response to increasing executive task demands in younger, but not in older adults, which may reflect a less responsive dopamine system in the face of cognitive challenge (Karlsson et al., 2009). All this evidence points to a greater need in older participants for more tyrosine substitution. We observed no significant differences in habitual daily tyrosine intake between the younger and older group but—as expected—a strong group difference in cognitive performance, and enhanced tyrosine intake by older adults might reduce this difference to some extent.

When exploring the association of tyrosine intake with the respective food categories of the food frequency questionnaire, we found the strongest correlation with the amount of meat products consumed in both the younger and older age groups.

A major limitation of the present study is that the assessment of dietary tyrosine took place on average two years before the cognitive assessment (mean difference in days 760, SD 442). However, we think that, because the period on which the dietary questionnaire focused was the last 12 months, the reports most likely reflect what participants eat on a regular basis. Furthermore, the nutrient intake results are based on observational data only, which strictly does not allow causal claims about the effects of tyrosine on cognition, and specifically does not preclude unobserved common causes of both cognition and nutrition that may explain the observed associations. For example, socio-economic status may be a common cause associated with both higher cognitive performance and higher use of nutritional supplements, better adherence to dietary rules, or lower fat intake (Hulshof et al. 1991). However, to account for these effects, we controlled for years of education, and this did not alter the present results. Still, our results are based on between-subject comparisons and only with intervention studies that longitudinally assess nutrition and cognition on a within-subject basis will we be able to exclude possible common causes, and we firmly encourage further work in this direction. Another clear limitation is that other amino acids such as tryptophan are highly correlated with tyrosine levels, which makes it difficult to ensure that the present effects are attributable solely to tyrosine. Future research may focus on investigating the covariance structure between different nutrients; that is, it would be worthwhile to investigate which nutrients are typically also ingested with a diet high in tyrosine. Another potential weakness of our study, brought to our attention by a thoughtful reviewer, is that assessing dieting behavior by means of a questionnaire is in itself an episodic memory test. This could explain why we not only observe the expected association between tyrosine intake and WM and Gf, but also with EM, which was not one of our core a priori hypotheses.

The present results show that, irrespective of the large age difference in cognitive performance, the relationship between tyrosine and the latent cognitive dimensions of WM, EM, and Gf is not different between younger and older participants. This underlines the generalizability of the association between habitual dietary tyrosine and cognitive function across age even with greatly varying levels of cognitive performance.
